# How does online social interaction promote students’ continuous learning intentions?

**DOI:** 10.3389/fpsyg.2023.1098110

**Published:** 2023-01-30

**Authors:** Shunan Zhang, ShaoPeng Che, Dongyan Nan, Jang Hyun Kim

**Affiliations:** ^1^Department of Interaction Science, Sungkyunkwan University, Seoul, Republic of Korea; ^2^Department of Human-Artificial Intelligence Interaction, Sungkyunkwan University, Seoul, Republic of Korea; ^3^School of Journalism and Communication, Tsinghua University, Beijing, China

**Keywords:** Danmu videos, online learning, motivations, hindrances, continuance intention

## Abstract

Learning from online videos using synchronized Danmu comments provides a co-learning experience. To explore the factors influencing learning with or without Danmu videos, an initial set of reasons and impediments was compiled based on a pilot study of 24 Chinese university students with learning experience using Danmu videos. Three hundred such students were surveyed to determine the factors that influence their motivations and hindrances with regard to using Danmu videos. The potential predictors of users’ continuance intentions were also examined. The results showed that the frequency of using Danmu videos is related to the continuous learning intention. Seeking information, social presence, and perceived entertainment motivate learners and positively impact their intention to continue learning using Danmu videos. Hindrances such as information pollution, attention failure, and visual obstacle were found to be negatively associated with learners’ continuance intention in the long run. Our findings provided constructive suggestions for addressing the issue of dropout rates, and novel ideas for future studies were proposed.

## 1. Introduction

As of 2022, the rapid growth of online learning has significantly drawn the attention of researchers. Online education can be accessed in numerous ways. Researchers have examined MOOCs to explore approaches for learning on platforms such as Coursera (Young, 2012; Rossi and Gnawali, 2014; Bates, 2019). Some researchers have explored video-sharing website-assisted learning, such as YouTube (Kruse and Veblen, 2012; Tan, 2013; Buzzetto-More, 2015). Researchers have also focused on learning through social media (Mondahl and Razmerita, 2014), such as Facebook (Kabilan et al., 2010; Fewkes and McCabe, 2012; Prescott et al., 2013), Twitter (Lowe and Laffey, 2011; Rinaldo et al., 2011; Evans, 2014), and WeChat (Jin, 2018). In China, however, there is another special type of learning video, namely Danmu video, which is widely enjoyed by young people.

Previous studies have considered the features of Danmu and its impact in videos. For example, the language features of Danmu in videos have raised concerns among several linguists (Zhang and Cassany, 2019). Scholars have also focused on the impression of Chinese democracy created *via* discussions on Danmu videos (Yin and Fung, 2017) and a virtual heterotopia to oppose social norms, control, and consumerism (Gu, 2017; Chen, 2021). Computer scientists have explored motivations of Internet users to watch videos using Danmu (Chen et al., 2017). However, although learning with Danmu videos is increasingly becoming a new form of online learning, studies that determine the reasons for the enthusiastic use of Danmu videos as a learning resource are limited.

Despite the appeal of providing education with very flexible time and space boundaries, some real concerns about online learning exist. Many authors state that traditional MOOCs (Massive Open Online Courses) are not very effective due to the lack of student–teacher and student-to-student interaction; however, when opportunities for interaction and teacher responsiveness are added, the effectiveness can be improved significantly (Ma et al., 2022). Moreover, previous research has shown that high interaction rates are positively associated with students’ intention to continue learning (Fang et al., 2019; Zhang et al., 2022). In Danmu, which uses a new type of commenting system, people can interact with each other during the learning process. With increased interactions, we predict that the use of Danmu will have a positive impact on the improvement of the student continuity.

In this regard, the purpose of the current study is twofold. First, we aim to explore the reasons why Danmu videos are used or not used for learning. Furthermore, as dropout rates from online learning have been a concern in the academia, we address how learning with Danmu videos can affect students’ intention to continue learning online.

## 2. Theoretical frameworks

### 2.1. Danmu videos

The unique learning experience offered by Danmu videos includes the following:

#### 2.1.1. Relative real-time interaction

In contrast to traditional commenting systems where comments are presented separately from the video content, Danmu comments work in real time while the video streams (Zhang and Cassany, 2019). During the learning process, learners can post comments based on their thoughts, feelings, questions, etc., synchronous with the video content. Compared with the separate discussion pages provided by platforms such as MOOC, Danmu can reduce the time and energy for users in taking notes, asking questions, and searching for answers in the forum later, while watching the videos.

#### 2.1.2. “Pseudo-synchronous” learning

As observed by Johnson (2013), Danmu comments provide users with “pseudo-synchronous” co-learning experience. Participants are encouraged to discuss precise, specific, and real-time information with other learners using Danmu promptly, instead of generic impressions and *post-hoc* remarks. Learning with a large number of Danmu comments helps learners perceive the presence of others.

#### 2.1.3. Learning with more information

Interesting and informative comments may appear when a video is viewed by a large audience. By turning on the Danmu commenting system, anyone watching the video can view the displayed information, providing the viewer with information beyond the content of the video.

### 2.2. Bilibili

Bilibili is one of the most popular Danmu video websites in China (Lin et al., 2018). As a comprehensive video-sharing website, users can upload, share, view, and comment on various video clips. Although originally created for entertainment purposes, Bilibili has emerged as a major platform to learn, providing a wealth of learning resources for 183 million young people studying using Bilibili. Therefore, in this study, Bilibili was selected as a case study to explore the learning behaviors of users with Danmu comments.

### 2.3. Social interaction in online learning

Social interaction is regarded as a fundamental way to meet social needs (Turk et al., 2022). Some existing studies have highlighted the significance of social interaction and have provided useful theoretical insights into whether and how social interaction may impact online learning (Cho and Cho, 2014; García-Peñalvo, 2014; Al-Rahmi et al., 2018). For example, Kurucay and Inan (2017) found that learner–learner interaction in an e-learning environment plays an important role in improving learning performance, and that students who interact with their peers appear to perform better than those who do not. Similarly, Molinillo et al. (2018) showed that both learner–instructor and learner–learner interactions influence learners’ emotional involvement and boost their active learning. Social interaction is thought to be vital for generating critical thinking and boosting learning outcomes, because it can improve social presence and minimize feelings of isolation among learners (Zhang et al., 2017; Fang et al., 2018). Furthermore, as Cobb (2008) indicated, social interaction is highly correlated with student satisfaction and perceived learning.

Peer interaction not only allows students to have meaningful conversations with their peers and gives them opportunities to practice high order thinking skills, which can ultimately improve learning, but it also helps students feel connected to each other within the community and increases their sense of social presence. In this study, we focus on the interactions that take place during an online learning process using the Danmu commenting system. Learners can interact in real time during the video presentation and also read and interact with the comments posted by viewers who have previously watched and commented on the same Danmu video.

### 2.4. Continuance intention

Despite the rapid development and numerous advantages of online learning, student retention in online courses remains as something challenging to achieve, with high dropout rates causing anxiety among instructors and administrators (Chiu et al., 2005; Roca et al., 2006). Comprehensive exploration of the factors that affect students’ continuance intention and how to improve it (Wu and Chen, 2017; Yang et al., 2017; Joo et al., 2018) have been carried out. Danmu, an innovative comment system, has brought about different experiences to students’ learning compared to traditional learning. Can the appearance of the Danmu contribute more toward improving students’ persistent intentions? This study focuses on the characteristics of Danmu and explores how the Danmu commenting system affects the continuance learning intention of learners.

In light of the above, the present study aims to explore the following research questions:

RQ1: What are the motivations for learning with Danmu videos?

RQ2: What are the hindrances for learning with Danmu videos?

RQ3: How does the Danmu commenting system influence people’s continuance intention in online learning?

## 3. Method

### 3.1. Pilot study: Collection of factors influencing university students’ behaviors of learning with/without Danmu videos in Bilibili

Owing to a lack of previous research, a pilot study was conducted to understand the comments and opinions of university students on Danmu videos in Bilibili. We used focus groups for data collection to facilitate the generation of different perspectives through group interactions (Krueger, 2014).

#### 3.1.1. Participants

Twenty-four participants were recruited by sending invitations *via* WeChat. All the participants were university students and had more than 1 year of learning experience with Danmu videos in Bilibili. Based on their responses to a five-point Likert scale of Danmu video viewing frequency (every time, often, sometimes, occasionally, hardly ever) on Danmu video viewing frequency, 12 participants were classified as frequent users (learning Danmu videos every time or often in Bilibili), whereas the rest were classified as infrequent users (learning using Danmu videos occasionally, or hardly ever). To stimulate discussion from different perspectives, all the participants were divided into four focus groups, with each group consisting of three students learning with Danmu videos frequently and three infrequent users.

#### 3.1.2. Procedure

Studies involving all the focus groups were held in Zoom calls. Before the focus group study began, the facilitator explained the purpose of the study and each participant signed an informed consent form (ensuring participant anonymity and confidentiality). Participants were then given 5 min to review their experience of learning with Danmu videos in Bilibili and complete a questionnaire about their basic information. In each focus group study lasting 50–60 min, the video and audio were recorded and retained with the permission of the participants.

#### 3.1.3. Questions

The research team created a semi-structured question guide based on the suggested focus group methods (Krueger, 1997), aiming to identify the factors influencing university students’ attitudes toward the Danmu commenting system when using Bilibili as a learning resource. Focus group discussions were initiated through opening and introductory questions, thus helping the participants to start a conversation on the topic naturally, after which a few transition and key questions were asked to lead the group to the major section of the conversation and to keep the emphasis on the study’s goal, which was to discover factors that influence students’ learning behaviors with or without Danmu in Bilibili. The facilitator followed an outline of questions throughout the focus group discussion to gain additional in-depth information on the topic, and a few additional questions were also asked flexibly to facilitate free discussion among the students as the following.

*Opening question: Where are you from and what is your major?*

*Introduction: What are your reasons for using Bilibili as a learning resource?*

*Transition: What are your views regarding the comments posted on Danmu videos?*

*Key questions: Do you like learning using Danmu comments in Bilibili?*

*What factors motivate you to learn with Danmu comments?*

*What factors motivate you to turn off Danmu comments when you are learning?*

*What type of Danmu comments would you prefer to see?*

*Ending: Do you have any additional comments toward learning, with or without Danmu in Bilibili? We are exploring interactive ways and experiences of online learning, and we would be grateful for any further suggestions, if you have any.*

Audios of the four sessions were fully recorded and transcribed into text format. When analyzing the data, all the members of the research team reviewed the transcripts collectively, exchanged thoughts, and discussed the key issues discovered. To form opinions about the data and ensure the reliability of the data interpretation, the members through the aforementioned stage three times. Finally, the factors were grouped into the following categories.

Motivations of learning with Danmu comments in Bilibili are as follows:

*Social presence*. Being able to study together online was one of the main reasons why most of the participants turned on the Danmu comments while studying in Bilibili. Online learning can be a lonely experience (Labrague et al., 2021), but Danmu provided them with a strong feeling of learning and connection with peers, thus overcoming the problem of isolation in an online learning environment.

*Perceived usefulness*. In the reviews, all the participants highly emphasized the value of Danmu with regard to the fact that useful information can be obtained *via* such videos. Individuals expressed the effectiveness of Danmu videos in helping them understand the learning content, especially open-minded content. “The views of others expressed through Danmu always bring me inspiration.” In addition, they mentioned that if learners encounter difficulties when learning, they can always ask questions in Danmu and will usually always receive a reply.

*Perceived enjoyment*. Learning is a boring process; however, a significant amount of interesting content is shown in Danmu reviews, which stimulates participants’ interest in learning; this makes them more willing to turn on Danmu and enjoy the fun of learning in Bilibili.

Reasons for learning without Danmu comments in Bilibili are as follows:

*Distracted attention*. The majority of the participants highlighted that Danmu comments were a distraction during learning, and they therefore turned them off. Danmu comments can sometimes contain irrelevant, repetitive, and low-value information (e.g., negative personal emotions and disagreement statements between learners in terms of differing opinions or ideas). Therefore, students’ attention can easily be drawn to such information, which may, in turn, reduce the effectiveness of online learning.

*Visual obstacle experience*. The fonts and colors of the Danmu comments were perceived as unattractive by some participants. In terms of viewing experience, some participants mentioned that the Danmu comments ruined the esthetic feel of the learning platform and even obscured the content of the original video; this was found to be very distracting by the aforementioned participants.

### 3.2. Survey: Identification of the factors affecting learning with Danmu in Bilibili

#### 3.2.1. Participants

The participants in this study were university students who had learned from Danmu videos in Bilibili. For data collection, we conducted an online survey for this study using a professional research panel provided by Credamo (a well-known online questionnaire company in China). Three hundred valid questionnaires were collected from Chinese university students who had experienced learning using Danmu videos in Bilibili. All participants filled out an informed consent form. The demographic information of the participants is presented in Table 1.

**Table 1:** Demographic statistics of participants (*N* = 300).

Measures	Items	Frequency	Percentage (%)
Gender	Male	90	30
	Female	210	70
Educational level	Undergraduate	250	83.3
	Graduate	50	16.7
Discipline	Social science and humanities	158	52.7
	Natural science	142	47.3
Frequency of learning with Danmu videos	Always	52	17.3
	Often	117	39
	Sometimes	55	18.3
	Occasionally	62	20.7
	Hardly ever	14	4.7

#### 3.2.2. Instruments

The questionnaire was divided into four parts. In Part 1, we collected demographic information (i.e., gender, educational level, and discipline) and asked questions related to the frequency of learning with Danmu videos in Bilibili. Parts 2 and 3 were about the factors influencing learning with Danmu videos. Part 4 was designed to examine the students’ continuous intention to learn using Danmu videos. The items used for the measurement of continuous learning intention were extracted and modified from Fu et al. (2020).

All items in part 2,3,4 was scored on a 5-point Likert scale (Joshi et al., 2015). To remove the language barrier, the questionnaire was translated into Chinese by professional translators and checked by two native Chinese speakers to ensure that the content was comprehensible and free from ambiguity. Then, the draft questionnaire was pilot tested with 15 students from different universities who learned using Danmu videos in Bilibili. Some items were modified according to their suggestions.

## 4. Result

### 4.1. Descriptive analysis of gratifications, hindrances, and continuous intention of learning with Danmu videos

As shown in Table 2, when comparing the reasons for studying with Danmu videos, students reported higher levels of social presence (Mean = 3.98, SD = 0.63), followed by gaining useful information through Danmu (Mean = 3.90, SD = 0.82). Finally, there is the perceived interestingness that comes with Danmu (Mean = 3.79, SD = 0.71).

**Table 2:** Factors of gratifications of learning using Danmu videos.

Factors	Items	Mean	Standard deviation
Perceived usefulness	Danmu brings me useful information	3.89	0.95
	Danmu helps me to access information effectively	4.25	0.79
	Danmu gives me more inspiration	3.96	0.83
	Danmu helps me to better understand the content of the learning videos	3.95	0.89
	Sometimes the content of Danmu helps to answer my questions	3.92	0.9
	Mean = 3.79, SD = 0.71		
Perceived entertainment	Danmu is funny	3.94	0.99
	Danmu makes me feel relaxed	3.85	0.96
	Danmu makes learning more fun	3.92	0.92
	Danmu brings me joy even when the content of the learning videos is boring	3.89	0.95
	Mean = 3.9, SD = 0.82		
Social presence	Danmu makes me feel like a member of a learning community	3.88	0.84
	Danmu makes me think I am studying with my classmates	4.25	0.79
	When my thoughts are consistent with the Danmu, it gives me a sense of resonance	3.96	0.83
	Danmu creates an atmosphere and opportunity for me to interact and communicate with other learners	3.89	0.78
	Danmu reduces my feelings of isolation	3.95	0.89
	Danmu gives me a sense of companionship when I study	3.92	0.9
	Mean = 3.98, SD = 0.63		

As shown in Table 3, information pollution, which addresses issues of spam in Danmu (repetitive, negative, emotional discharge, not relevant, no value messages) ranked first (Mean = 3.83, SD = 0.75). Attention failure ranked second (Mean = 3.13, SD = 0.91). Visual obstacle (Mean = 3.00, SD = 0.93) ranked last.

**Table 3:** Hindrance factors in learning with Danmu videos.

Factors	Items	Mean	Standard deviation
Attention failure	Danmu diverts myself from the watching experience of the original video	3.53	1.09
	Danmu distracts me from my studies	3.18	1.12
	Danmu is not conducive to me being fully engaged when I study	2.79	1.06
	It is difficult for me to engage in my work when my views are not in line with those in Danmu	2.88	1.12
	I am easily distracted from my study time by Danmu	3.3	1.14
	Mean = 3.13, SD = 0.91		
Visual obstacle	The format of Danmu is unattractive	2.71	0.9
	Danmu obscures the content of the original video	2.91	1.13
	The colorfulness Danmu is unattractive	3.36	1.12
	Mean = 3, SD = 0.93		
Information pollution	There is information in Danmu that is not relevant to learning	3.94	0.85
	There are repetitive messages in Danmu	4.21	0.75
	There are emotional messages in Danmu	3.68	1.01
	There are negative messages in Danmu	3.47	1.04
	There is no valuable information in Danmu	3.85	0.92
	Mean = 3.83, SD = 0.75		

As shown in Table 4, students express a high intention to continuously learn with Danmu videos (Mean = 3.53, SD = 0.92). The Cronbach’s alpha was 0.92, which meets the recommendation level (Tavakol and Dennick, 2011).

**Table 4:** Continuous learning intention.

Factors	Items	Mean	Standard deviation
Continuous learning intention (Fu et al., 2020)	In the future, I will continue to use Danmu videos for learning	3.61	0.942
	I will recommend learning using Danmu videos to others	3.58	1.111
	I will open Danmu comments as I learn	3.72	0.975
	I will continue to interact and communicate with other learners in Danmu	3.44	1.094
	I would be happy to use Danmu videos in my future studies	3.29	1.1
	Mean = 3.53, SD = 0.92		
	Cronbach’s alpha = 0.92		

### 4.2. Reliability and validity tests

A principal component factor analysis with varimax rotation was conducted on the 28 items to check the validity of measurements and determine whether the key dimensions of motivation and hindrance in learning with Danmu videos were consistent with the focus group results.

Table 5 shows the Kaiser–Meyer–Olkin test (KMO) and Bartlett’s test results. As shown in Table 5, the KMO value was more than 0.5, and the Bartlett’s test was significant (*p* < 0.001). The validity test of the questionnaire is satisfactory (Tabachnick et al., 2007).

**Table 5:** Kaiser–Meyer–Olkin test (KMO) and Bartlett’s test.

Kaiser–Meyer–Olkin measure of sampling adequacy		0.943
Bartlett’s test of sphericity	Approx. Chi-square	4970.513
	Df (no. of degrees of freedom)	325
	*p*-value (Sig.)	0

Table 6 shows the rotated component matrix. In line with the focus group results, a total of six factors were generated. Items with a factor loading of above 0.3 can be retained. However, social presence1, social presence5, and social presence6 were removed owing to cross loading (Goni et al., 2020). In total, the six components generated accounted for 70.31% of the total variance, indicating that the model was a good fit (Montoya and Edwards, 2021).

**Table 6:** Rotated component matrix.

	1	2	3	4	5	6
Information seeking1	0.751					
Information seeking2	0.735					
Information seeking3	0.567					
Information seeking4	0.685					
Information seeking5	0.559					
Perceived entertainment1		0.711				
Perceived entertainment2		0.722				
Perceived entertainment3		0.664				
Perceived entertainment4		0.665				
Social presence1						
Social presence2			0.558			
Social presence3			0.713			
Social presence4			0.765			
Social presence5						
Social presence6						
Information pollution1				0.742		
Information pollution2				0.686		
Information pollution3				0.813		
Information pollution4				0.795		
Information pollution5				0.755		
Visual obstacle1					0.633	
Visual obstacle2					0.738	
Visual obstacle3					0.686	
Attention failure1						0.597
Attention failure2						0.609
Attention failure3						0.788
Attention failure4						0.704
Attention failure5						0.764

For the reliability test, a value of Cronbach’s alpha above 0.7 is considered to have very good internal consistency (Tavakol and Dennick, 2011). The reliability of these factors was accepted, with Cronbach’s alpha values ranging from 0.76 to 0.88 (Table 7).

**Table 7:** Cronbach’s alpha test results.

Factors	Cronbach’s alpha
Information seeking	0.87
Perceived entertainment	0.88
Social presence	0.76
Information pollution	0.87
Visual obstacle	0.87
Attention failure	0.88

### 4.3. Continuance intention of learning with Danmu videos

Regression analyses were conducted to explore the factors influencing students’ intention to continue learning from Danmu videos. Preliminary operations were performed to confirm the assumptions of independence, singularity, and multicollinearity (all variance inflation factors were <4). In both regression analyses, demographic differences, such as gender, educational level, grade, age, discipline, and use frequency, were entered first to control for the possible influence of these factors. The factors contained in motivations and hindrances were then analyzed. The results are presented in Table 8.

**Table 8:** Effect on the continuance intention of learning using Danmu videos.

	*R* square	Beta	*p-*Value	VIF	Durbin–Watson
Stage1	0.547				1.804
Gender		−0.79	0.326	1.049	
Educational level		0.016	0.906	1.941	
Grade		−0.18	0.722	2.299	
Age		0.242	0.126	1.293	
Discipline		−0.006	0.497	1.079	
Using frequency		0.739	0	1.032	
Stage2	0.811				1.967
Gender		−0.075	0.155	1.059	
Educational level		0.053	0.544	1.972	
Grade		0.021	0.529	2.357	
Age		0.085	0.405	1.305	
Discipline		−0.001	0.920	1.09	
Using frequency		0.207	0.000	2.02	
Information seeking		0.342	0.000	3.038	
Perceived entertainment		0.2	0.000	2.524	
Social presence		0.199	0.000	2.222	
Information pollution		−0.116	0.006	1.772	
Visual obstacle		−0.1	0.015	2.64	
Attention failure		−0.098	0.013	2.309	

In terms of demographic differences, as the value of *p*-values corresponding to gender (*p* = 0.326), education level (*p* = 0.906), grade (*p* = 0.722), age (*p* = 0.126), and discipline (*p* = 0.497) were all greater than 0.05, there is no significant effect on sustained intention to learn using Danmu videos. However, as the value of *p* is much smaller (*p* = 0.000), using frequency has a significant positive influence toward the continuance intention of learning with Danmu videos.

Regarding motivations and hindrances, information seeking (*β* = 0.342, *p* < 0.001), perceived entertainment (*β* = 0.20, *p* < 0.001), and social presence (*β* = 0.199, *p* < 0.001) positively influence students’ continuous learning intentions. There is a negative relationship between information pollution (*β* = −0.116, *p* < 0.05), virtual obstacle (*β* = −0.1, *p* < 0.05), attention failure (*β* = −0.098, *p* < 0.05), and continuous learning intentions. In total, these seven factors explain 81% of students’ continuous learning intentions, which exceeds the recommended value (Cameron and Windmeijer, 1997).

**Figure 1:**
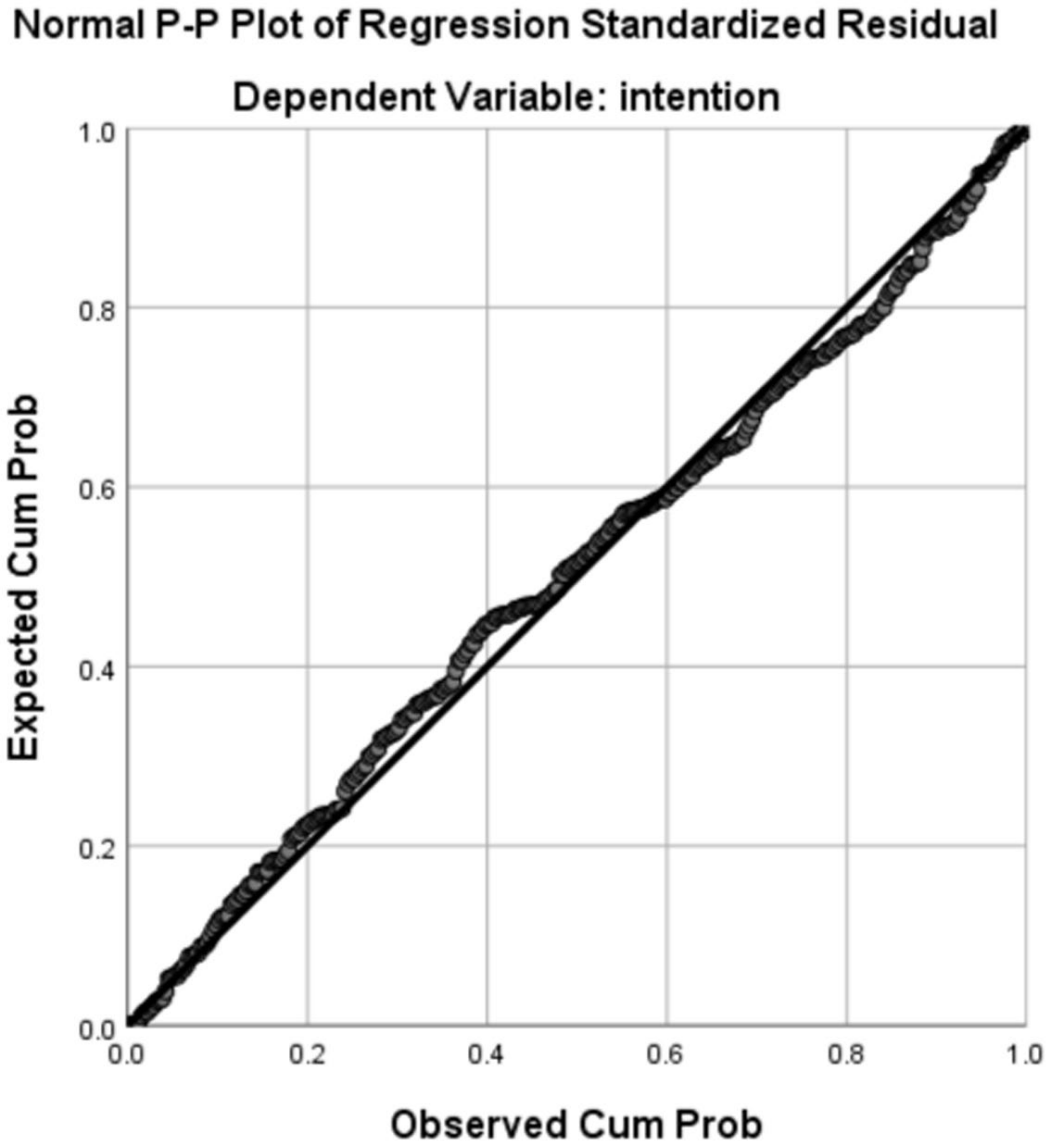
Normal distribution.

To ensure that there was no multicollinearity between the variables, multicollinearity diagnosis was performed. As shown in Table 8, the VIF values were all less than 5 (Mansfield and Helms, 1982). Additionally, the residuals followed a normal distribution as shown in Figure 1. The Durbin-Watson value was around 2, which satisfies the recommended value (Farrar and Glauber, 1967). Therefore, we concluded that there was no multicollinearity between our independent variables and our findings are reliable.

## 5. Discussion

Online learning platforms such as *Coursera* and *edX* provide their users with separate pages, such as discussion forums or discussion pages, to interact and communicate (Lin et al., 2018). However, the separation of learning content from commentary not only limits student interaction but also negatively affects students’ sense of social presence and continued learning intentions (Wong et al., 2015). Real-time, horizontal, text-based Danmu is a product of social interaction, and it encourages students to remain engaged, interested, and committed throughout their studies. Our findings revealed that Danmu-driven social interaction provides users with advantages such as seeking additional information, perceived entertainment, and enhanced perceived social presence. All these factors are positively attributed to students’ continuous learning intentions.

### 5.1. Demographic differences

There was no difference in the evaluation of continuance intention to learn using Danmu videos by research respondents in terms of demographic variables such as age, educational level, grade, and discipline, which is in agreement with the results of previous studies (Almahamid and Rub, 2011). However, interestingly, there is a clear positive relationship between the frequency of use of Danmu videos and the intention to learn with it consistently.

### 5.2. Gratifications of learning using Danmu videos

Information seeking is the strongest positive predictor of users’ continuance intention to learn from Danmu videos. It is the embodiment of meeting utilitarian needs based on uses and gratification theory (Bradley, 1974). Utilitarian characteristics are also in line with the concept of perceived usefulness in the technology acceptance model (TAM), both emphasizing practicality (Venkatesh and Bala, 2008). Perceived usefulness was positively correlated with online learning continuance intention. This is consistent with previous studies (Saadé, 2007; Chang and Tung, 2008; Lee, 2008; Tang et al., 2014). Danmu offers a unique way of seeking information and acquiring knowledge. First, Danmu helps users access information more effectively. By learning with background knowledge or answers to doubts, students can better understand the teaching content. Second, opinions expressed by others in Danmu stimulated students’ thinking and inspired them. More interestingly, as the video is played more often, Danmu may provide more information. This means that over time, learners will find different and more information through Danmu.

Social presence is the second major predictor that motivates people to persistently learn using Danmu videos in Bilibili. This result is in line with previous research on the positive predictors of continuance intentions (Hayashi et al., 2004; Smith, 2006; Smith and Sivo, 2012). Online learning is a long-lasting experience. Students who study online have higher levels of loneliness than those who study in traditional classes (Gillett-Swan, 2017; Kaufmann and Vallade, 2020; Savci et al., 2022). Loneliness is a negative factor that influences online learning in various academic contexts (Vakoufari et al., 2014; Ali and Smith, 2015). However, learning synchronously with Danmu comments creates an atmosphere of companionship, thereby reducing loneliness with the increase of social presence.

Perceived entertainment is the third factor that predicts students’ positive ongoing learning intentions. This is consistent with previous studies (Gao et al., 2020; Zhou et al., 2022). How to make learning more interesting has been a topic of interest for researchers. As mentioned by Jiang (2014) even if the content of the video is boring, the sense of enjoyment that Danmu brings positively influences the user’s video viewing experience. Although self-learning is boring, Danmu helps learners to relax even if the learning content is boring.

### 5.3. Hindrances in learning using Danmu videos

Information pollution is the strongest negative predictor of continuance learning intention. Information pollution emphasizes the fact that Danmu comments contain a lot of repetitive, irrelevant, and even negative information. As cognitive load theory (Sweller, 2011) reveals, the presence of factors unrelated to learning during the learning process may place a cognitive load on the learner and thus negatively affect the learner’s learning outcomes. Cognitive load has been widely demonstrated when using new media resources for learning (Krancher et al., 2019; Zheng et al., 2022), and therefore, instruction should be designed to minimize the cognitive load on learners.

Attention failure is the second negative predictor of continuance intention. The Internet has provided people with easier access to a variety of resources, including learning and entertainment. During online learning, students are likely to be drawn to other information that pops up in the Internet, which prevents them from concentrating and negatively affects their learning (Lin et al., 2022). While the Danmu commenting system provides a co-learning environment for students, there is also a lot of “noise” that distracts students from learning process.

Visual obstacles negatively affect students’ ongoing learning intentions. Visual obstacles are when there are too many Danmu and the content of the video is obscured by them. Even though Danmu can be set to display position, transparency, etc., some users still perceive Danmu to be a visual obstacle.

## 6. Conclusion and implications

The extreme popularity of Danmu videos provided participants with a “pseudo-synchronous” learning experience. Based on a literature review, pilot study, and survey of 300 participants, this study explored the motivations, hindrances, and continuance intention of learning using Danmu videos. Our findings revealed three motivations for learning using Danmu videos: seeking additional information more easily and feeling a sense of social presence and perceived entertainment. The three major hindrances are visual obstacle, attention failures, and information pollution. Concerning continuance intention, motivations are obviously positive factors, with information seeking ranked first, followed by social interaction and entertainment. Information pollution, attention failure, and visual obstacles pose threats to participants’ continuance intention. Additionally, usage frequency also contributes to students’ continuous learning intention with Danmu videos.

### 6.1. Theoretical implications

To the best of our knowledge, this study is the first attempt to investigate the motivations, hindrances, and continuance intention of learning using Danmu videos. These results underline the special value of the Danmu commenting system in information acquisition, social presence, and perceived entertainment. In an innovative social interaction way, Danmu not only helps students better understand the video content through the provision of additional information but also makes outstanding contributions to solving the problems of social presence and perceived entertainment in online learning. In addition, from the perspective of persistent intention, motivation to learn using Danmu is also a positive predictor of continuance intention, which may provide solutions for addressing the issue of high dropout rates.

### 6.2. Practical implications

This study also provides practical implications for promoting students’ use of Danmu videos in the learning process. Learning using Danmu videos provides the desired co-learning experience with easier information seeking and higher level of social presence and entertainment value. However, even frequent users indicate that information pollution, visual obstacle, and attention failure influence their studies to some extent. For Danmu developers, balancing the positive and negative values of Danmu has become the key to improving the role of Danmu. Furthermore, in future, Danmu is expected to be combined with more learning platforms. Therefore, more efforts should be focused on the integration of Danmu and video learning platforms, such as MOOCs, to explore its influence on continuance intention and behavioral intention.

### 6.3. Limitations and suggestions for future research

This research has some limitations, although it has provided insight into the continuance intent of Danmu videos in the learning process. First, the results are based only on students’ opinions. Therefore, the conclusions about the effect of each observed factor are subjective. Modalities such as big data can be considered to obtain more objective research results. Second, the participants recruited in this paper cover all provinces of China, but the study does not consider the differences between provinces. Future research could consider the differences between region, ethnicity, etc. Lastly, this study only focuses on Chinese students, emphasizing the practical, social, and entertainment value of Danmu. Future research should consider whether the application of Danmu in other countries can have the same effect.

## Data availability statement

The raw data supporting the conclusions of this article will be made available by the authors, without undue reservation.

## Ethics statement

Ethical review and approval was not required for the study on human participants in accordance with the local legislation and institutional requirements. The patients/participants provided their written informed consent to participate in this study.

## Author contributions

SZ: conceptualization, research design, implementation, writing, and editing. SC: conceptualization, writing, and editing. DN: writing and editing. JK: conceptualization, research design, writing, and editing. All authors contributed to the article and approved the submitted version.

## Funding

This paper was supported by SKKU Global Research Platform Research Fund, Sungkyunkwan University, 2022.

## Conflict of interest

The authors declare that the research was conducted in the absence of any commercial or financial relationships that could be construed as a potential conflict of interest.

## Publisher’s note

All claims expressed in this article are solely those of the authors and do not necessarily represent those of their affiliated organizations, or those of the publisher, the editors and the reviewers. Any product that may be evaluated in this article, or claim that may be made by its manufacturer, is not guaranteed or endorsed by the publisher.
